# Harnessing Standing Sound Waves to Treat Intraocular Blood Cell Accumulation

**DOI:** 10.3390/mi15060786

**Published:** 2024-06-15

**Authors:** Avraham Kenigsberg, Shany Shperling, Ornit Nagler-Avramovitz, Heli Peleg-Levy, Silvia Piperno, Alon Skaat, Ari Leshno, Hagay Shpaisman, Noa Kapelushnik

**Affiliations:** 1 Department of Chemistry, Institute of Nanotechnology and Advanced Materials, Bar-Ilan University, Ramat Gan 5290002, Israel; avikening@gmail.com (A.K.); ornitavr@gmail.com (O.N.-A.);; 2 Sackler Faculty of Medicine, Tel Aviv University, Tel Aviv 6997801, Israelaskaat11@gmail.com (A.S.); kapelushniknoa@gmail.com (N.K.); 3 Goldschleger Eye Institute, Sheba Medical Center, Tel Hashomer, Ramat Gan 5262000, Israel

**Keywords:** acoustic manipulation, intraocular particles, standing waves, anterior chamber

## Abstract

Certain ocular conditions result from the non-physiological presence of intraocular particles, leading to visual impairment and potential long-term damage. This happens when the normally clear aqueous humor becomes less transparent, thus blocking the visual axis and by intraocular pressure elevation due to blockage of the trabecular meshwork, as seen in secondary open-angle glaucoma (SOAG). Some of these “particle-related pathologies” acquire ocular conditions like pigment dispersion syndrome, pseodoexfoliation and uveitis. Others are trauma-related, such as blood cell accumulation in hyphema. While medical and surgical treatments exist for SOAG, there is a notable absence of effective preventive measures. Consequently, the prevailing clinical approach predominantly adopts a “wait and see” strategy, wherein the focus lies on managing secondary complications and offers no treatment options for particulate matter disposal. We developed a new technique utilizing standing acoustic waves to trap and direct intraocular particles. By employing acoustic trapping at nodal regions and controlled movement of the acoustic transducer, we successfully directed these particles to specific locations within the angle. Here, we demonstrate control and movement of polystyrene (PS) particles to specific locations within an in vitro eye model, as well as blood cells in porcine eyes (ex vivo). The removal of particles from certain areas can facilitate the outflow of aqueous humor (AH) and help maintain optimal intraocular pressure (IOP) levels, resulting in a non-invasive tool for preventing secondary glaucoma. Furthermore, by controlling the location of trapped particles we can hasten the clearance of the AH and improve visual acuity and quality more effectively. This study represents a significant step towards the practical application of our technique in clinical use.

## 1. Introduction

Particulate matter can disperse and accumulate within the anterior chamber from different causes such as hyphema, pseodoexfoliation syndrome, pigment dispersion syndrome and uveitis [1,2,3,4]. These pathological particles can lead to various complications due to interactions with intraocular tissues and disruption of their function. The particles can cause visual disturbance resulting from impairment of the aqueous humor (AH) clarity, such as commonly seen in the acute phase of hyphema, pigment dispersion or uveitis [5,6,7]. Visual disturbance can also result from the long-standing presence of particles that lead to “staining” of the corneal endothelium or lens capsule. Intraocular particles in the AH can also lead to obstruction of the trabecular meshwork (TM) and disruption of the primary drainage pathway, causing a rise in IOP and the development of SOAG [8]. Although there are medical and surgical treatments to deal with these particle-related complications [9], effective preventive measures are lacking. 

The applications of acoustics in the field of medicine can be divided into diagnostic use, mainly by auscultation and ultrasound, which is common in almost all fields of medicine as well as in ophthalmology [10], and therapeutic use such as in high-intensity focused ultrasound (HIFU), which uses high-frequency acoustic waves to selectively thermal ablate part of the ciliary body, intending to reduce IOP and treat glaucoma [11]. Other applications of acoustic waves in medicine were investigated, for example, the use of low-energy surface acoustic waves to prevent microbial biofilm formation on medical devices [12] and the use of acoustic waves to dissect or fragment tissues [13].

**Figure 1:**
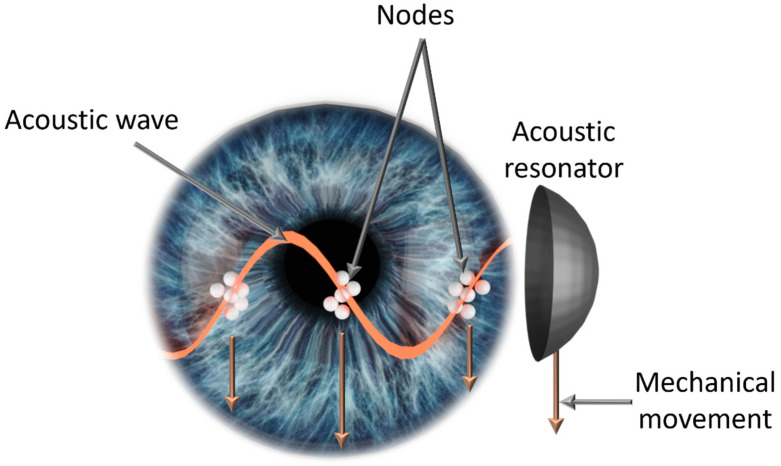
Illustration of interocular particles trapped and directed utilizing the acoustic method presented in this manuscript. An acoustic transducer generates standing acoustic waves to guide intraocular particles toward nodal areas. Subsequent mechanical movement of the transducer induces a downward pull on the particles.

In 2022, Leshno et al. reported a new technique for trapping intraocular particles in the anterior chamber using standing acoustic waves [14]. The method demonstrated effective particle localization in both in vitro and ex vivo experiments, without inducing any damage to the cornea. Here, we hypothesize that this technology may be harnessed as a preventive treatment for these particle-related syndromes. By controlling and manipulating floating particles (Figure 1), they can be condensed and moved to a desired location, as previously shown for various materials systems [15,16]. This kind of intervention would help clear the visual axis and simultaneously reduce the extent of the interaction of pathological particles with the trabecular meshwork.

The purpose of the current study is to test the feasibility and utility of using the acoustic manipulation technique to clear the visual axis and reduce particle accumulation within the trabecular meshwork using in vitro and ex vivo models of hyphema.

## 2. Materials and Methods

We developed a device intended to trap and manipulate suspended particles within the anterior chamber. Our device was tested in vitro using polystyrene (PS) particles injected into the anterior segment of an artificial eye model and using red blood cells injected into an ex vivo porcine eye. The device and models are explained in the following sections.

### 2.1. Intraocular Manipulation Device

Acoustic resonators (model: QN9-26NC/30, diameter: 9 mm, focal point: 30 mm) with a resonance frequency of 2.6 ± 0.1 MHz were purchased from Siansonic (Beijing, China). The resonator was driven by a signal generator (Siglent (Solon, OH, USA) SDG 5162) with a continuous sine wave at an amplitude of 0–10 volt peak-to-peak (V_pp_) with 50 Ohm resistance to create pressure waves inside the reservoir. A power amplifier (20 kHz–50 MHz) was used for amplification to a maximum of 30 Vrms. Experiments were conducted at 29.5 ± 0.5 Vrms unless stated otherwise. 

**Figure 2:**
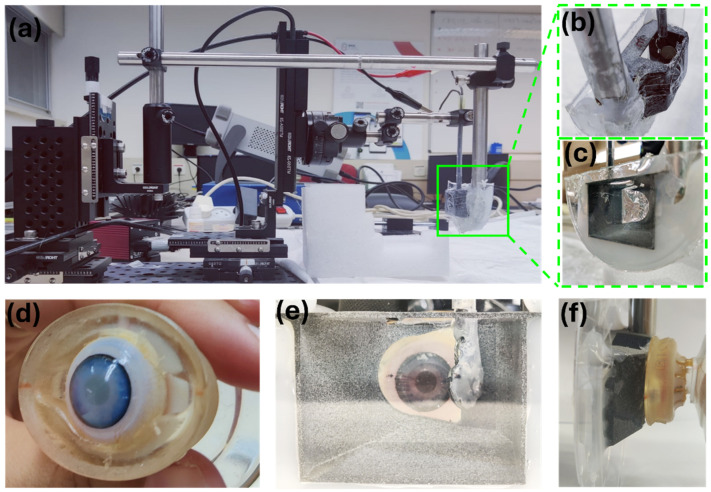
(**a**) Side view of the intraocular manipulation device. (**b**) Rear view (eye side) of the 3D-printed reservoir housing the acoustic resonator. (**c**) Front view (camera side) of the 3D-printed reservoir housing the acoustic resonator. (**d**) Eye model depicting PS particles within the anterior chamber. (**e**) Front view of an eye model attached to the 3D reservoir. (**f**) Side view of an eye model attached to the 3D reservoir.

The acoustic resonator was connected to a computer-controlled motorized stage (KDC101 K-Cube™ Stepper Motor Controller, Thorlabs (Newton, NJ, USA)) capable of movement along the Z-axis. Additionally, the stage allows for manual adjustments along the X-axis and Y-axis and tilt in two axes (Figure 2). A 3D polyamide-printed reservoir filled with water was used to ensure acoustic coupling between the resonator and the treated eye. A transparent polycarbonate plate was attached to the printed reservoir on one side and a thin transparent polyethylene film on the other side (Figure 2b,c). Transparency allowed for the visualization of the process while the thin film allowed for good contact between the eye and water inside the reservoir (Figure 2e). The acoustic resonator was placed inside the reservoir, and acoustic waves traveled through water and the thin polyethylene film before reaching the cornea. Standing acoustic waves were generated by wave reflections inside the cornea. As the cornea is spherical, sections with a length of n times the wavelength always existed, thus forming a standing acoustic wave that enabled the trapping of suspended particles. Manipulation of the particles was performed by controlled movement of the motorized stage (for additional details, refer to Figure S1).

### 2.2. In Vitro Model 

Anterior segment models (MING-SC, Bioniko (Miami, FL, USA)) were used to imitate the movement of intraocular microparticles when subjected to an acoustic field generated by our acoustic manipulation device. The ability to generate standing acoustic waves inside the anterior chamber was examined.

To simulate particulate matter, a polystyrene (PS) microsphere suspension was injected into the anterior chamber of the in vitro eye models. The suspension was prepared by diluting white 10 µm (Alfa Aesar) 2.5 wt% polystyrene microsphere suspension with DI water to a final concentration of 1.5 wt%. 

The model’s cornea was covered with 1.4% Hydroxyethylcellulose (Celluspan, Dr. Fischer) to improve acoustic coupling and attached to the device’s thin transparent polyethylene film. 

**Figure 3:**
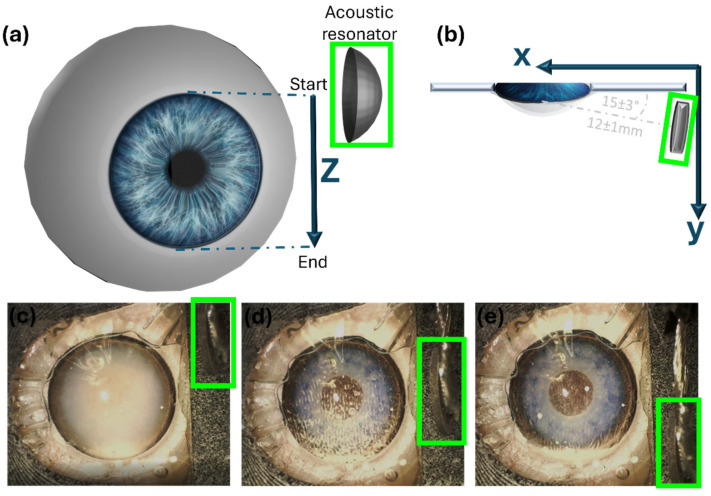
(**a**) Illustration of the acoustic resonator (green outline) movement along the Z-axis during one run and of the (**b**) top view of the acoustic resonator and eye positions during a run. (**c**) AC eye model after PS injection (before the run). (**d**) AC model during the run. (**e**) AC model after the run ended.

The resonator center was initially positioned 12 ± 1 mm from the cornea center of the eye model at a 15 ± 3° angle relative to the reservoir’s plane (Figure 3). Subsequently, the resonator was moved upward to the upper corneal limbus of the model (Figure 3c), and the acoustic wave was then activated. The motorized stage was then moved downward along the Z-axis (Figure 3d) from the upper limbus to the lower limbus (run—distance: 12 mm, speed: 0.1 mm/s). At the lower limbus height, the wave was deactivated (Figure 3e), and the resonator moved upward to the starting point at the upper limbus. Note that our experimental design should direct particles to the lower limbus, in synergy with gravitational force.

To evaluate the effect of the acoustic field on the microparticles’ movement, 3 runs were applied on each eye model, and the process was recorded using a Dino Lite Edge Digital Microscope camera (SV1). Reference samples were used to evaluate the gravitational effect. Polystyrene suspension was injected into the control samples, which were placed at the same position as the test models for 30 min without activating the acoustic wave, solely influenced by gravity. The experiment was repeated 10 times.

**Figure 4:**
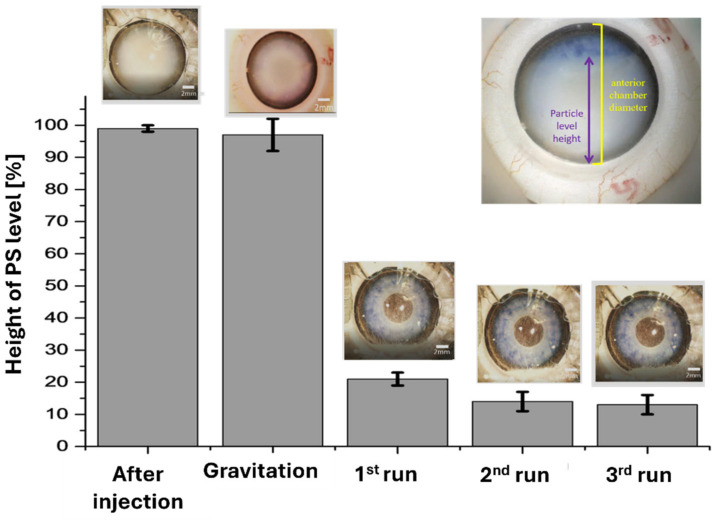
Average change in polystyrene (PS) height with gravitation only (30 min), as a reference, and with acoustic treatment (3 runs).

To quantitatively assess the extent of evacuation, we analyzed the PS particle distribution along the visual axis using microscope images. A quantitative estimate was derived through image analysis using ImageJ software (v1.54f), which utilized the change in brightness of PS to detect the particle level height (refer to Figure 4). The ratio of the particle level height to the chamber diameter was calculated and multiplied by 100 to yield the height of PS as a percentage.

### 2.3. Ex Vivo Model

Porcine eyes were retrieved from the local abattoir within 4 h post-mortem. To imitate the accumulation of red blood cells (RBCs) within the anterior chamber (hyphema), 0.1 mL of blood suspension was injected into an ex vivo porcine anterior chamber by means of a clear corneal incision with a 30 G needle prior to attachment of the eyes to the acoustic device. Blood suspensions were prepared by diluting blood with a saline solution of 0.9% NaCl 1:5 by volume. 

After the injection of blood, the eye was left in a vertical position undisturbed for about 20 min to monitor blood sedimentation, solely influenced by gravity. The cornea was covered with 1.4% Hydroxyethylcellulose and attached to the device’s thin transparent polyethylene film. The device’s resonator center (along the Z-axis) was located at the upper corneal limbus height and the acoustic wave was activated. The motorized stage was moved downward along the Z-axis from the upper limbus to the lower limbus (run).

Three runs were applied on each treated eye, and the process was recorded using a Dino Lite Edge Digital Microscope camera. Some eyes were used as control and were not treated with the acoustic waves. Both the treated and reference eyes were immersed in 4% paraformaldehyde for 48 h and then sent for histologic evaluation. Histologic analysis and comparison of the upper and lower part of the AC angle was performed by assessing the number of pixels representing RBC using ImageJ software to determine the upper/lower ratio; the sum of the pixels representing RBC was divided by the total number of pixels of each image. The Mann–Whitney U test was used for comparison between the upper and lower parts of the angle. A *p* value of <0.05 was considered statistically significant. The data were analyzed with SPSS software version 25.0 (SPSS Inc., Chicago, IL, USA). A thermal imaging camera (Therm-App TH^®^, Opgal Optronic Industries Ltd., 184 Carmiel, Israel) was placed in front of the ex vivo eyes to capture and record heat changes during the treatment. 

**Figure 5:**
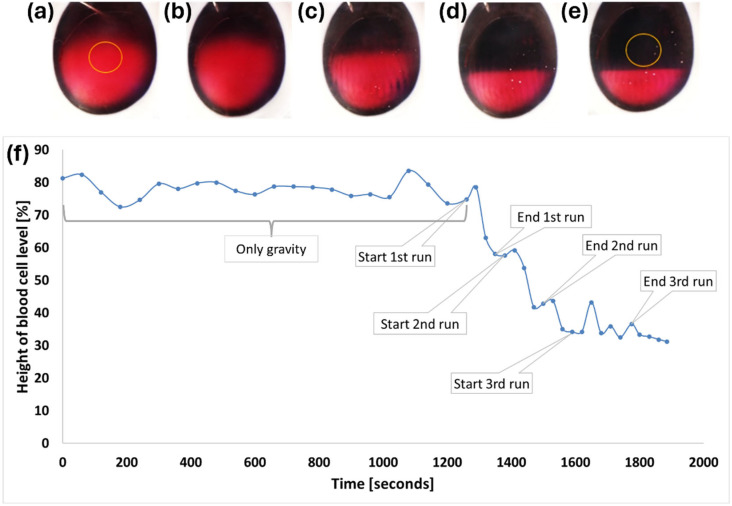
Microscope images of a vertically positioned porcine eye with human blood. The visual axis is marked by an orange circle: (**a**) initial state. (**b**) After 21 min without acoustic waves. Minimal sinking is observed. (**c**) End of the first run. (**d**) End of the second run. (**e**) End of the third run. (**f**) Changes in blood cell height (as a percentage of the anterior chamber height) due to gravity and during treatment in a single experiment.

To quantitatively assess the extent of evacuation, we analyzed the blood cell particle distribution along the visual axis using microscope images. A quantitative estimate was derived through image analysis using ImageJ software, which utilized the change in red amplitude to detect the blood cell level height (refer to Figure 5f). The ratio of the blood cell level height to the chamber diameter was calculated and multiplied by 100 to yield the height of blood cells as a percentage.

## 3. Results

### 3.1. In Vitro Model

The PS particles were observed to accumulate in stripe-like patterns at the nodes of the standing acoustic wave. By moving the resonator parallel to the stripes, particles were driven towards specific locations—our automated resonator movement directed the particles to the lower part of the AC. The results of our experiment are presented in Figure 4, which depicts the change in PS particle concentration as a function of runs using an amplitude of 29 Vrms. Our findings show that after only one run, we were able to clear more than 70% of the anterior chamber height. We repeated the protocol two additional times (second and third runs) and significantly improved clearance to more than 82% of the anterior chamber height. Beyond three runs, we did not observe significant changes in PS height. 

### 3.2. Ex Vivo Model

Twelve bovine enucleated eyes were included in this study. All were injected with blood suspensions. Seven were treated with the acoustic resonator, and five were used as a control. Both macroscopic clearance of the AC from blood and histological differences between the upper and lower part of the AC angle were assessed. 

As indicated in Figure 5a,b, gravity had a minimal effect on blood subsidence, and only minor sedimentation occurred. By conducting three consecutive runs of the acoustic wave, moving from the top to the bottom of the AC, the hyphema was effectively directed to the lower portion (Figure 5c,d) and cleared the visual axis (Figure 5e). The blood cell level height as a percentage of the anterior chamber height over time (the entire process) can be observed in Figure 5f.

**Figure 6:**
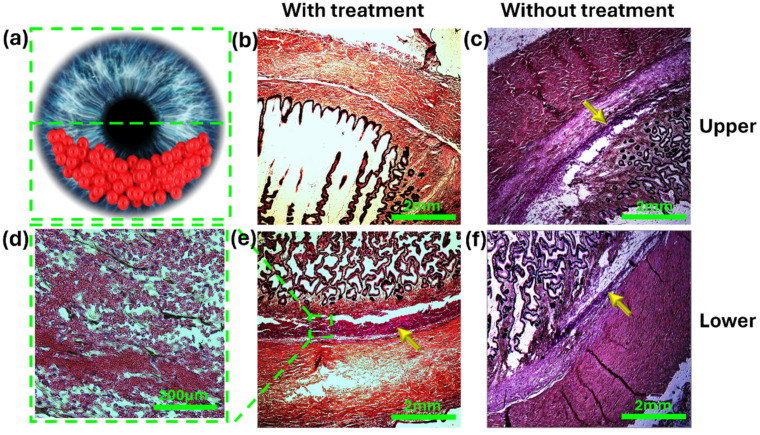
Histology of the angle in treated and non-treated eyes. (**a**) Illustration of the AC following treatment with an acoustic wave to inferiorly displace the RBCs. (**a**,**b**) Histology sections of the upper portion of the angle showing minimal numbers of red blood cells in the treated eye (**b**), and a large number of red blood cells in the non-treated eye (**c**, yellow arrow). (**d**) High magnification of the lower angle of a treated eye showing a large number of condensed red blood cells. (**e**,**f**, yellow arrow) Histology sections of the lower angle showing a larger number of red blood cells in the treated eye (**e**) compared with the non-treated eye (**f**).

Figure 6 shows a representative histologic microscope image of the AC angle with and without treatment. In the treated eyes, we observed a significant number of red blood cells (RBCs) in the lower part compared with the upper part of the angle (*p* = 0.03, Mann–Whitney U test). In the non-treated eyes, the number of RBCs was non significantly higher in the upper part of the angle (*p* = 0.84, Mann–Whitney U test).

The eyes treated with acoustic waves exhibited a significant disparity between the upper and lower angle portions (upper–lower ratio: 0.4, *p* value = 0.005, student *t*-test), whereas in the controls, there was no significant difference between the two portions (upper–lower ratio: 1.43, *p* value = 0.24). This demonstrates the efficacy of our approach in preventing accumulation in the upper portion of the TM. The maximum temperature increase recorded was 3 °C. Microscopic assessment and histologic analysis revealed no visual damage resulting from the acoustic waves.

## 4. Discussion

The purpose of the current study was to test the feasibility of an acoustic manipulation technique to clear the visual axis and reduce particle accumulation within the trabecular meshwork in hyphema. We found that this technique can be used to relocate particles effectively in the anterior chamber in both artificial and ex vivo eye models. Specifically, we were able to rapidly clear the visual axis as well as protect the superior segments of the TM from particle accumulation.

In this study, we refined our previous method [14] of employing acoustic standing waves to trap intraocular particles. Unlike our earlier approach, which relied on a cylindrical resonator and lacked the ability to relocate particles as desired, we now employ a single resonator, utilizing corneal reflection. With computer-controlled resonator movement, we achieve precise, non-invasive manipulation of particles to predetermined locations. The angle and distance of our concave resonator from the AC were chosen for ease of implementation in our current setup. However, a separate study that simulates and experimentally verifies the optimal effects of the position and angle of the resonator relative to the eye is beyond the scope of this paper and will be conducted independently.

To date, commonly accepted treatment modalities for hyphema consist of intensive monitoring, topical treatment for lowering intraocular pressure, mydriatics to prevent iris adhesions and topical corticosteroids to prevent associated inflammation. However, none of these interventions has been proven to be effective [9]. In refractory cases, surgical intervention is indicated to prevent serious irreversible complications such as corneal staining and secondary glaucoma [17,18]. Currently, there is no non-invasive method to facilitate the clearance of the anterior chamber from blood cells. Consequently, patients afflicted with hyphema due to diverse reasons (e.g., traumatic, uveitis–glaucoma–hyphema syndrome, iris neovascularization, etc.) are forced to wait patiently for the red blood cells to disperse and clear the visual axis before their vision is restored. 

Our experimental results indicate that the acoustic manipulation technique may have practical clinical applications for treating hyphema. Using our device, we were able to manipulate and control intraocular particles within the anterior chamber. Trapping and moving these particles downwards was found to be highly effective in condensing the particles compared with gravitation alone. By guiding the particles downward, we successfully cleared most of the anterior chamber. This not only facilitated the clearance of the visual axis but also enhanced the visualization of intraocular structures. In a clinical setting, this kind of treatment is expected to both improve the patient’s visual acuity as well as the physician’s ability to examine the posterior portion of the eye. 

The ability to control the pathological intraocular particles in a non-invasive manner, as shown in this study, can also be used to prevent damage to the TM. The homogenic scatter inside the anterior chamber of these particles obstructs significant areas of the TM and thus causes inefficient drainage of AH and, eventually, IOP rise and secondary optic nerve damage [8]. Histological analysis of the anterior chamber angle demonstrated a significant reduction in particles in the upper portion of the angle. These results suggest that acoustic manipulation might be used to reduce obstruction of the TM by particulate matter non-invasively and has the potential to prevent the development of SOAG. By consolidating particles into a single location, we may sacrifice a small portion of the lower angle, which would be affected by gravity regardless. However, this strategy minimizes the risk of particles interacting with the TM in the majority of the angle, ensuring an ample drainage area to maintain proper intraocular pressure.

Though our results are promising, this study has some limitations. First, this is an ex vivo experiment; thus, it is hard to conclude the effect in vivo in eyes that move and change their location constantly. Although we conducted an ex vivo shaking test and observed minimal drift of blood cells (Figure S4), further in vivo experiments are needed to evaluate the efficacy of our model on a living eye in a more clinical setting. Our hypothesis is that in vivo, the natural flow of aqueous humor through the angle will keep the manipulated particles in the lower part of the angle, where we intentionally manipulated them to localize, allowing the upper part of the angle to remain open and clean with no particle obstruction. Furthermore, clearing hyphema using our novel method has potential in vivo since the normal eye in humans is upright, thus allowing synergism with the gravitational power, which also moves the particles downward, enabling the clearance of the visual axis. Another limitation of our study is the difficulty in evaluating the safety of the acoustic manipulation in an ex vivo model. While our current study revealed no damage to the ex vivo bovine eye, it is essential to note that further investigation is required to ascertain any potential in vivo ocular damage.

## 5. Conclusions

We have introduced a novel method for trapping and directing intraocular particles to specific locations without causing any damage to ocular tissue in ex vivo experiments. This technique has the potential to be used to treat hyphema by clearing the visual axis and facilitating visual improvement and better visualization of intraocular structures. As sound waves would have a similar effect on various types of particulate matter dispersed in the AH (including pigment particles [14]), clearing the upper part of the angle may also be a preventive treatment for secondary glaucoma by protecting the majority of the angle from interacting with the particles. In vivo studies are required to evaluate the effect of eye motions on long-term efficiency and to rule out any safety concerns. Furthermore, the development of a computational model to elucidate the propagation characteristics of acoustic waves within the anterior chamber is essential for optimizing the orientation and trajectory of the resonator.

## 6. Patents

Leshno, A.; Skaat, A.; Kenigsberg, A.; Shpaisman, H. An Ocular Acoustic Device and a Method Thereof, PCT/IL2021/050862.

## Data Availability

All data generated or analyzed during this study are included in this article. Further inquiries can be directed to the corresponding authors.

## Supplementary Materials

The following supporting information can be downloaded at: https://www.mdpi.com/article/10.3390/mi15060786/s1, Video S1: In vitro experiment demonstrating arrangement and movement of polystyrene microparticles using acoustic waves generated by the acoustic resonator and mechanical movement of the resonator. Supporting Information: Acoustic apparatus—more details; Experimental investigation using a 1 MHz resonator; Impact of post-treatment eye movement.

## Abbreviations

The following abbreviations are used in this manuscript:

SOAG	secondary open-angle glaucoma
PS	polystyrene
IOP	intraocular pressure
AH	aqueous humor
TM	trabecular meshwork
HIFU	high-intensity focused ultrasound
V_pp_	volt peak-to-peak
