# Supplementary material for: Harnessing Standing Sound Waves to Treat Intraocular Blood Cell Accumulation
Source: Micromachines (Basel). 2024 Jun 15;15(6):786. doi: 10.3390/mi15060786 (PMC11205910; doi:10.3390/mi15060786)
Supplement: Supplementary file 1 [file micromachines-15-00786-s001.zip › micromachines-3043829-Supporting information_micromachines.pdf]

Supporting Information

Harnessing Standing Sound Waves to Treat Intraocular Blood Cells Accumulation

Avraham Kenigsberg [3], Shany Shperling[1,2], Ornit Nagler-Avramovitz[3], Heli Peleg-Levy [3], Silvia Piperno [3], Alon Skaat [1,2], Ari Leshno[1,2,*], Hagay Shpaisman [3,*] and Noa Kapelushnik [1,2]

1 Sam Rothberg Glaucoma Center, Goldschleger Eye Institute, Sheba Medical Center, Ramat-Gan, Israel
2 Sackler Faculty of Medicine, Tel Aviv University, Tel Aviv, Israel.
3 Department of Chemistry and Institute of Nanotechnology and Advanced Materials, Bar-Ilan University, Ramat Gan 5290002, Israel
* Correspondence: Hagay Shpaisman, PhD, Department of Chemistry and Institute of Nanotechnology and Advanced Materials, Bar-Ilan University, Ramat Gan 5290002, Israel. Email: hagay.shpaisman@biu.ac.il
Ari Leshno, MD PhD, Goldschleger Eye Institute, Sheba Medical Center, Tel Hashomer, 52621, Israel. E-mail: arileshno@gmail.com

Acoustic apparatus – more details

The electric signal traverses from the signal generator, through the amplifier, and then to the resonator (Figure S1a) via the electrical leads. The delicate resonator is positioned within a polymeric tube (Figure S1b), with the electrical leads also housed inside. The polymeric tube is capable of rotation along the Z-axis and is connected to the metallic holder of the acoustic resonator, which itself can rotate along the Y-axis. Movement along the Z-axis, allowing for upward and downward motion, is facilitated by a motorized stage controlled by the motorized stage controller (Figure S1c and S1d, respectively). The acoustic resonator

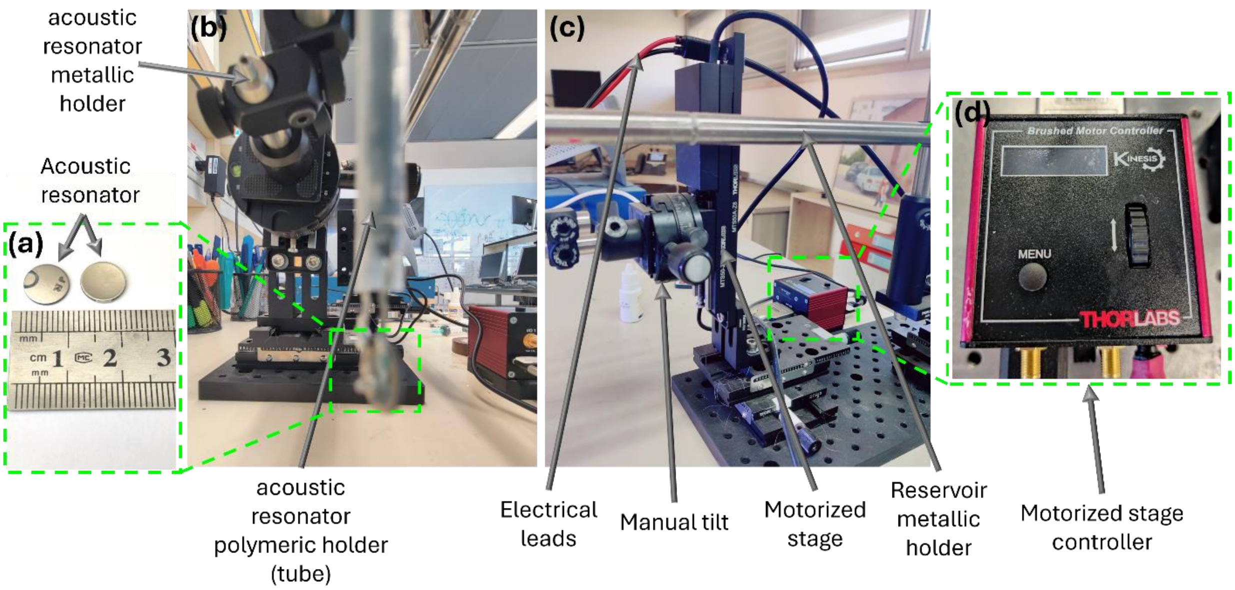

Figure S1. (a) The acoustic resonators (both sides) not connected to leads. (b) Image showing the resonator connected to electrical leads held by the polymeric tube. (c) Side view showing the motorized stage in the Z direction and manual control in other directions. (d) Motorized stage controller.

is housed within a 3D-printed reservoir (refer to Figure 2b in the manuscript), featuring a transparent section that facilitates visual observation, enabling the recording of the procedure. The 3D-printed reservoir is connected to a metal bar that can be manually moved along the X, Y, and Z axes.

Experimental Investigation Using a 1MHz Resonator

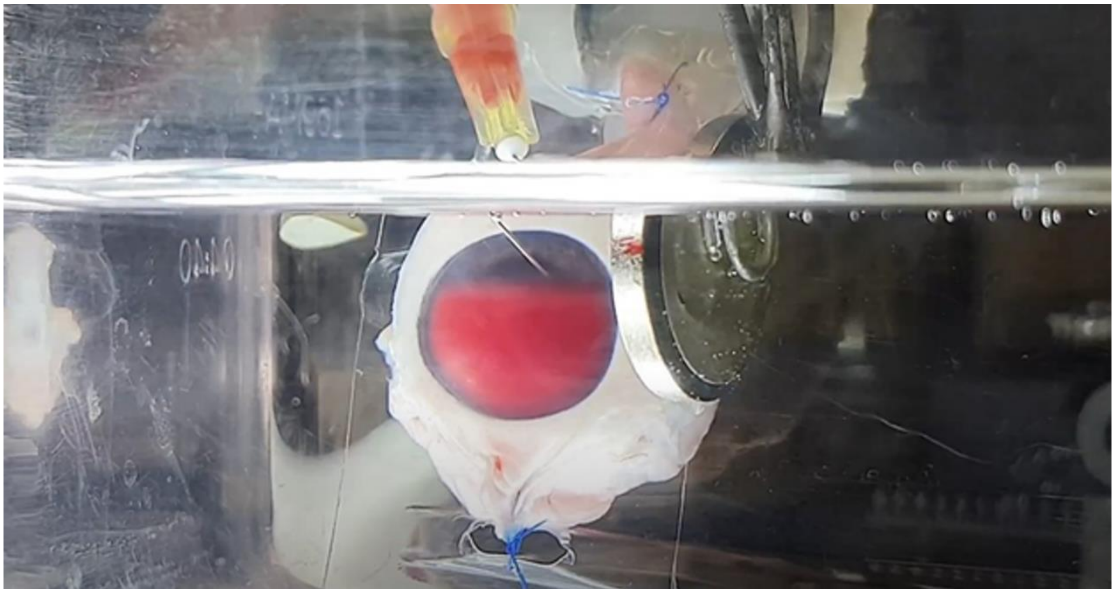

Figure S2. The acoustic resonator and ex-vivo porcine eye during blood injection inside plastic vessel filled with DI water.

Trapping and directing PS particles and blood cells were also tested using a 1 MHz, 20 mm diameter, and 35 mm focal point concave resonator (Siansonic Technology Co., Ltd, model QN20-10C/35). However, due to the larger size of the resonator, instead of utilizing a printed reservoir as depicted in the manuscript, both the eye model or the porcine eyes and the acoustic resonator were inserted into a transparent vessel filled with DI water (refer to Figure S2). All the orientations and movements of the resonator were identical to those depicted in the manuscript. A Dino Lite Edge Digital Microscope was used to document our experiments. ImageJ software was utilized to measure the anterior chamber and visual axis clearance compared to reference conditions. As the PS particles appear white in water, the brightness intensity was analyzed and served as a measure to assess changes in PS coverage. The pupil (visual axis) and the anterior chamber appear darker before the injection of PS dispersion. PS particles highly reflect light, and as they fill the anterior chamber, they cover the underlying parts with a white layer. Acoustic waves are used to aggregate and manipulate PS particles to specific locations away from the visual axis. Reference images were taken before the activation of the acoustic waves, thereby neutralizing changes in lighting conditions and shooting angles. This analysis was conducted three times for each intensity, as shown in Figure S3. The most efficient evacuation was achieved at the highest amplitude measured (29 Vrms).

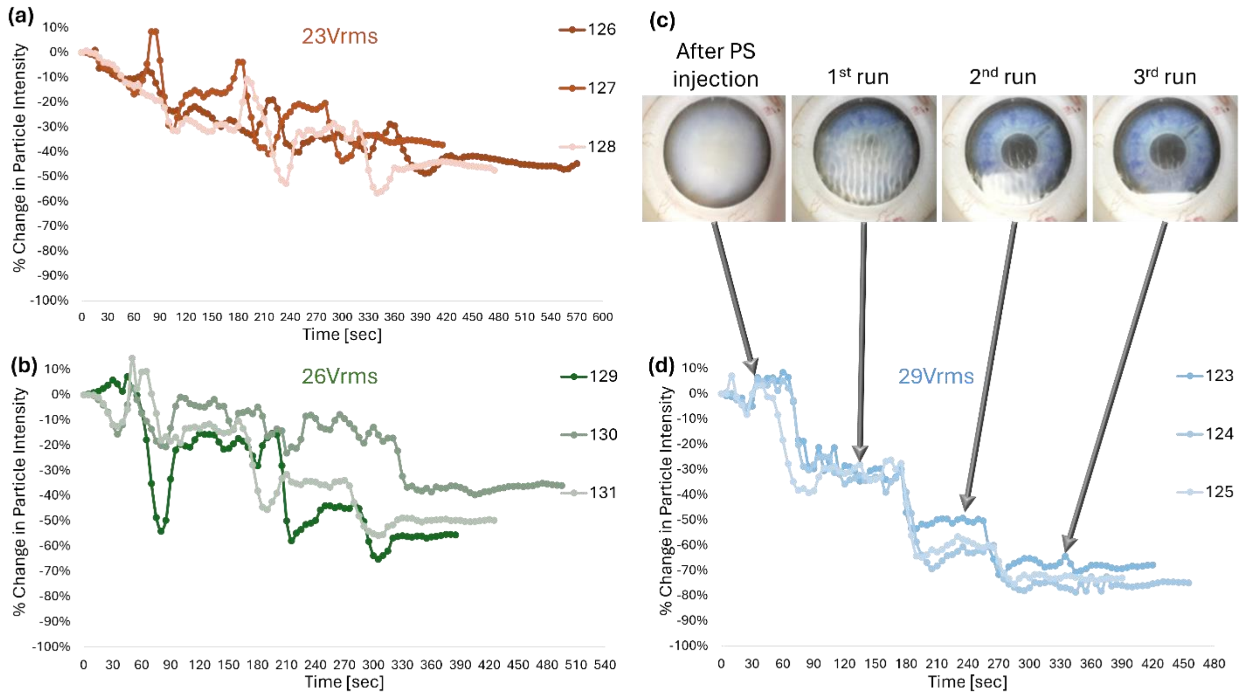

Figure S3. Change in brightness of a model eye pupil over time during PS manipulation using acoustic waves (3 "runs") at varying amplitudes with a 1MHz resonator. (a) 23 Vrms, (b) 26 Vrms and (d) 29 Vrms. (c) Images of the model eye pupil captured at different times during polystyrene (PS) manipulation using acoustic waves.

The Impact of Post-Treatment Movement

To evaluate whether the blood cells directed to the bottom part of the anterior chamber would remain there even after a subject moved freely, we simulated this behavior by shaking the eyes for 5 minutes at maximum speed of a Boekel Scientific Orbitron II Laboratory Mixer (model: 260250), as shown in Figure S4. We examined the blood distribution before and after shaking and found that only minimal amounts of blood drifted from the bottom part of the anterior chamber. However, in vivo trials need to be performed to determine the intervals at which this intervention with acoustic waves will need to be repeated.

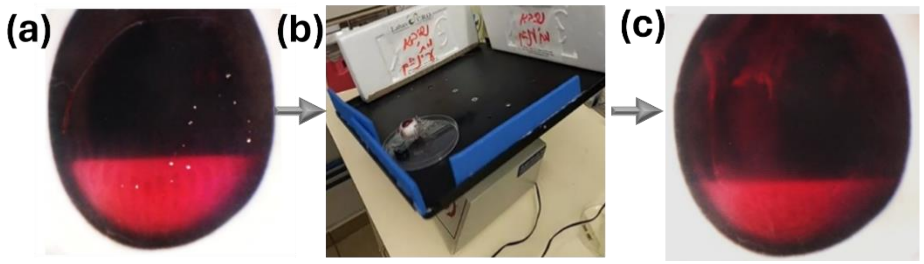

Figure S4. Microscope images of a vertically positioned porcine eye with human blood: (a) After acoustic manipulation before shaking, and (c) after 5 minutes of shaking. (b) Photograph of the device used to shake the porcine eye.
